# Supplementary material for: Targeting the SHP2 phosphatase promotes vascular damage and inhibition of tumor growth
Source: EMBO Mol Med. 2021 Jun 8;13(7):e14089. doi: 10.15252/emmm.202114089 (PMC8261520; doi:10.15252/emmm.202114089)
Supplement: Supplementary file 1 — Appendix [file EMMM-13-e14089-s004.pdf]

Appendix

Targeting the SHP2 phosphatase promotes vascular damage and inhibition of tumor growth

Yuyi Wang, Ombretta Salvucci, Hidetaka Ohnuki, Andy D. Tran, Taekyu Ha, Jing-Xin Feng, Michael DiPrima, Hyeongil Kwak, Dunrui Wang, Michael Kruhlak, Yanlin Yu and Giovanna Tosato

Table of Content

Materials and Methods

References

Figures with legends

Table

Supplementary Materials and Methods

Cells, cell culture and materials. HUVEC (Lifeline Cell Technology, Frederick MD; FC-0003) and HDMEC (Clonetics, CC-2543) were propagated as described (Kwak *et al*, 2016; Ritchey *et al*, 2019; Salvucci *et al*, 2009; Salvucci *et al*, 2015; Sanchez-Martin *et al*, 2018); HUVEC were used within passages 8 to 10 and HDMEC within passages 7 to 12. BMEC (Akt-1 activated murine bone marrow endothelial cells, a gift from Dr. Jason M. Butler (Department of Genetic Medicine, Weill Cornell Medical College, New York, USA) were propagated as described (Ritchey *et al.*, 2019). The murine melanoma B16F10 (ATCC CRL-6475), Lewis lung carcinoma LLC1 (ATCC CRL- 1642), plasmacytoma MOPC315 (ATCC TIB-23), colon adenocarcinoma MC-38 (Kerafast ENH204-FP Schlom J), and human embryonic kidney 293T (ATCC CRL-3216) lines were cultured in Dulbecco's Modified Eagle's Medium with 10% FCS. The human lung carcinoma A549 (ATCC CCL-185), mammary gland carcinoma Hs 578T (ATCC HBT-126), osteosarcomaG-292 (ATCC CRL-1423), NUGC3 gastric adenocarcinoma (JCRB 0822), colon carcinoma RKO (ATCC CRL-2577) and HT29 (ATCC HTB-38), and the murine mammary gland cancer 4T1 cell line (ATCC CRL-2539) were cultured in RPMI-1640 medium with 10% FCS. All cellslines tested *Mycoplasma*-negative but were not authenticated in the laboratory. SHP099 (MedChemExpress, HY-100388 and the Investigational Drugs Repository, DCTD, NCI) was solubilized in DMSO at 4mg/ml, aliquoted and stored at -80°C. Further dilutions were in PBS before addition to culture at varying concentrations 1-20μM). AMG386 (obtained from Amgen, Inc. under a Materials Cooperative Research and Development Agreement), was stored at -80°Cat 30mg/ml and used in culture at concentrations ranging from 0.01-10μM. Tofacitinib (Millipore Sigma, PZ0017), PD098059 (MedKoo Biosciences, 401680), AZD6244 (Selleckchem. S1008), and Trametinib (Selleckchem. S2673) were solubilized in

DMSO at 10mM, aliquoted and stored at -80°C. Tofacitinib was used in culture at 25nM or 50nM and PD098059 at 10μM.

Immunoprecipitation (IP) and Western blotting. Cells were washed with cold phosphate buffered saline (PBS) containing100nM sodium orthovanadate twice and room temperature PBSand harvested by scraping. Cell lysates were prepared with freshly prepared TNTG lysis buffer [50mM Tris-HCL (pH 7.4), 150mM NaCl, 1% Triton X-100, 10% glycerol, 2mM sodium orthovanadate, 1×phosphatase inhibitor cocktail 2 (Sigma, P5726) and 1×protease inhibitor cocktail (Thermo scientific, no. 78430)]. After incubation (1hr at 4 C°), lysates were centrifuged(10,000 g, 20 min) and supernatants stored at -80C°. For immunoprecipitation, cell lysates (250 mg) precleared with 25 ml protein G DynaBeads (Thermo Fisher, 10004D; 30 min at 4°C, rotating) were incubated with goat anti-EphB4 (1μg; R&D Systems AF3038) or goat IgG (1μg; R&D Systems AB-108-C) overnight at 4°C, rotating. The protein/antibody/beads complex was washed twice in TNTG buffer with high salt (500 nM NaCl), twice with TNTG buffer with low salt (150 nM NaCl). Protein was eluted from beads by incubation in sample buffer (1X LDS; Thermo Fisher, NP0007) containing 5% betamercaptoethanol (Sigma, M3148) at 100°C 5 min. Protein lysates and immunoprecipitates were separated by SDS/PAGE using NuPage 4-12% Bis-Tris gels (Thermo Fisher, NP0321 or NP0335) with either MOPS (Thermo Fisher, NP0001) or MES (Thermo Fisher, NP0002) running buffer and transferred to nitrocellulose membranes with iBot Gel Transfer system (Thermo Fisher, IB301001 or IB301002). After blocking with 5% bovine albumin fraction V (MP Biomedicals, 160069) for 90 mins, membranes were incubated with primary antibodies. Primary antibodies used include: from Cell Signaling Technologies, p- EphrinB (3481; 1:2,000); p-STAT3 (Tyr^{705}) (9145; 1:2,000); STAT3 (12640; 1;1000); p-Erk (Thr^{202}/Tyr^{204}) (9101; 1:1,000); Erk (p44/p42) (4695; 1:1,000); VE-cadherin (2500; 1:1000); STAT1 (9175;

1:1,000); p-STAT1 (Tyr^{701}) (9167; 1:1,000); STAT5 (94205; 1:1,000); p-STAT5 (Tyr^{694}) (9314; 1:1,000); p-AKT (Ser^{473}) (4060; 1:2,000), AKT (9272; 1:1,000), GAPDH (5174; 1:1,000); from Abcam, EphrinB2 (ab150411; 1:2000); TRP2 (ab74073; 1:2,000); from R&D Systems, mouse EphB4 (AF446; 1μg/ml); human/mouse p-TIE2 (0.5 μg/ml AF2720); from Santa Cruz Biotechnology, β-actin (sc-47778; 1:1,000). Secondary antibodies HRP-conjugated donkey anti-rabbit IgG (no. NA934V; 1:5,000), HRP-conjugated sheep anti-mouse IgG-Fc (no.NA931V; 1:5,000) (both from GE Healthcare Life Sciences) and IgG rabbit anti-goat (A27014;Thermo Fisher; 1:2,000). Bands were visualized using ECL prime kit (GE Healthcare Life Sciences, RPN2232) and captured digitally on a LAS4000 (GE LifeSciences). Bands fromWestern blotting were quantified by FIJI.

Immunofluorescence, imaging and image quantification. Immediately after collection, tissue samples were washed with cold PBS to remove blood and fixed with cold 4% PFA for 72 hours at 4 °C. Tissues were then processed by subsequent 24-hour incubations with PBS containing 10%, 20% and 30% sucrose and embedded in OCT. For immunostaining, 8 μm sections were layered onto glass slides and stored at -20°C. After thawing (room temperature for 15 min), sections were permeabilized with 1% Triton X-100/PBS (15 min), washed in PBS and incubatedwith Uni-Trieve solution (Innovex Biosciences, Richmond, CA, USA; NB325) at 75°C for 45 min. After washing three times with 1% Triton X-100/PBS, sections were blocked with blocking buffer (10% glycerol, 0.5% BSA, 0.4% Triton X-100 and 10% TBS) for 1 hour at roomtemperature. After rinsing in PBS, tissues were incubated overnight at 4°C with rat anti-mouse CD31/PECAM (Dianova, DIA-310; 1:100 or BD Pharmingen, 553370;1:100), mouse-anti- human CD31(Dako, JC70A, 1:100), rat anti-mouse Ly6G/Gr1 (BioLegend, 127618; 1:100), rabbit anti-pEphrinB (Cell Signaling Technologies, 3481; 1:100), rabbit anti-mouse NG2 (Millipore, AB5320; 1:200), rabbit anti-mouse Ki67 (Cell

Signaling Technologies, 9129; 1:100),rabbit anti-cleaved caspase-3 (Cell Signaling Technologies, no. 9579; 1:100), rabbit anti- human/mouse phospho-Tie2 (R&D Systems, AF2720; 1:100), rabbit anti-pSHP2 (Cell SignalingTechnologies, no. 3751; 1:100), rabbit anti-mouse collagen IV (Abd Serotec, 2150-1470; 1:100),rabbit anti-pSTAT3 (Cell Signaling Technologies, 9145; 1:100); rabbit anti-pErk (Cell Signaling Technologies, 9101; 1:100); PE rat anti-mouse Ly6C/Gr1 (BD Pharmingen, 560592; 1:200); Alexa Fluor 488 anti-mouse F4/80 (Biolegend, 123120, 1:200); rabbit anti-Angiopoietin 2/Ang2 (Abcam, 8452; 1:200); rabbit anti-FOXO1 (Cell Signaling Technologies, 2880; 1:100). Slides were then washed (three times with blocking buffer) and incubated with secondary antibodies: Alexa Fluor 488 donkey anti-rabbit IgG (Thermo Fisher; A21206), Alexa Fluor 594 donkey anti-rat IgG (ThermoFisher; A21209), Alexa Fluor 647 anti-rat IgG (A-21247) and Alexa Fluor 546 donkey anti-rabbit IgG (Life Technologies; A-10040) for 1 h, at 4°C. The slides were washed three times with blocking buffer, post-fixed with 4% PFA/PBS for 20 min at room temperature, washed three times with 1×TBS, and mounted with DAPI-containing mounting medium (Sigma,F6057).

Images were obtained by confocal microscopy (LSM 780; Carl Zeiss, Oberkochen, Germany) using ZEN software (Carl Zeiss). For imaging, extended field of view tile image of tissue sections were acquired using a Zeiss LSM780 laser scanning confocal microscope (Carl Zeiss, Oberkochen, Germany) equipped with a 20× plan-apochromat (N.A. 0.8) objective lens and 32-channel GaAsP spectral detector. Details of image acquisition and processing are found in Supplementary Materials and Methods. Three fluorescence emission channels for the respective sample labels, DAPI (em 410-480), Alexa Fluor 488 (em 490-555) and Alexa Fluor 546 (em 565-480 nm) were acquired. Confocal tile images were acquired with 0.19 mm x-y pixelsize and 5% image overlap. The images were stitched together using the Stitching

algorithm in the Zeiss Zen Blue (v.2.3) image processing software. Multiple regions ranging in size (1.1 mm^2 to 2.1 mm^2) were processed to count the number of DAPI-positive cells that were also positive for other markers (CD31, Ki67, cleaved caspase-3, P-TIE2, p-EphrinB, FOXO1, p-STAT3 and p-ERK). Necrotic tumor areas indicated by the absence of DAPI staining were excluded. Tumor tissues and areas to be imaged were chosen randomly. Image quantification was performed usingFIJI/Image J software (NIH) (Salvucci *et al.*, 2015). The tumor sections contain a significant number of red blood cells that have a broad autofluorescence spectrum across all three fluorescence channels. To eliminate red cell-derived autofluorescence, the red blood cells were segmented using the green fluorescence channel based on threshold intensity above background and size. An image mask corresponding to the red blood cells signal and a minimum roundness factor of 0.2 to account forred cell shape. This DAPI mask was then applied to the previously masked red channel image, with the resultant double-masked red channel image representing the red blood cell-free, DAPI- positive regions or cells. To measure collagen IV^+CD31^- sleeves in control and treated tumor tissues, we first segmented $CD31^+$ and collagen IV^+ areas by intensity threshold and then quantified the region areas. For $Ki67^+$ area/tumor area, size of $Ki67^+$ area was obtained the same way as we measured collagen IV, followed by the measurement of the corresponding tumor areasize. To measure the intensity of cleaved Caspase-3 in each endothelial cell, we generated a cell segmentation map from DAPI staining, which was masked using a threshold $CD31^+$ mask. We subsequently quantified the mean fluorescence intensity of cleaved Caspase-3. For the quantification of p-Tie2, p-EphrinB, p-STAT3, p-ERK or FOXO1 in each endothelial cell, we used the same method used to quantify cleaved caspase-3 in endothelial cells. Quantitative results are shown as % positive area and fluorescence intensity/cell.

References

Kwak H, Salvucci O, Weigert R, Martinez-Torrecuadrada JL, Henkemeyer M, Poulos MG, Butler JM, Tosato G (2016) Sinusoidal ephrin receptor EPHB4 controls hematopoietic progenitor cell mobilization from bone marrow. *J Clin Invest* 126: 4554-4568

Ritchey L, Ha T, Otsuka A, Kabashima K, Wang D, Wang Y, Lowy DR, Tosato G (2019) DLC1 deficiency and YAP signaling drive endothelial cell contact inhibition of growth and tumorigenesis. *Oncogene* 38: 7046-7059

Salvucci O, Maric D, Economopoulou M, Sakakibara S, Merlin S, Follenzi A, Tosato G (2009) EphrinB reverse signaling contributes to endothelial and mural cell assembly into vascular structures. *Blood* 114: 1707-1716

Salvucci O, Ohnuki H, Maric D, Hou X, Li X, Yoon SO, Segarra M, Eberhart CG, Acker-Palmer A, Tosato G (2015) EphrinB2 controls vessel pruning through STAT1-JNK3 signalling. *Nat Commun* 6: 6576

Sanchez-Martin D, Otsuka A, Kabashima K, Ha T, Wang D, Qian X, Lowy DR, Tosato G (2018) Effects of DLC1 Deficiency on Endothelial Cell Contact Growth Inhibition and Angiosarcoma Progression. *J Natl Cancer Inst* 110: 390-399

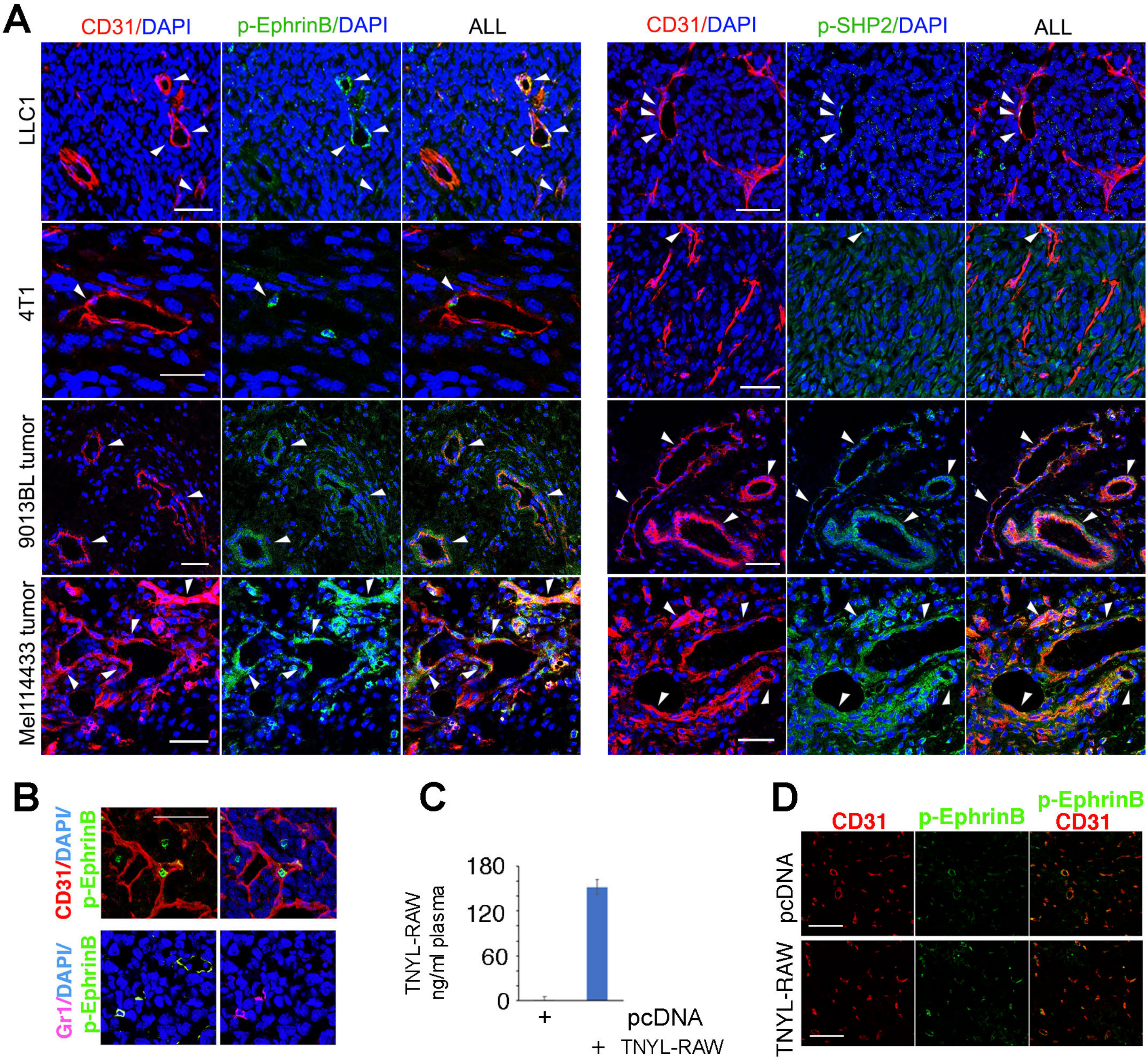

Appendix Figure S1

A p-EphrinB and p-SHP2 in CD31-positive tumor vessels of the indicated tumors from mice. Arrowheads point to p-EphrinB$^+$/CD31$^+$ and p-SHP2$^+$/CD31$^+$ cells.

B Gr1-positive myeloid cells in 4T1 tumor tissue are p-EphrinB-positive.

C TNYL-RAW peptide levels in the circulation of 5 mice transduced with control or TNYL-RAW expression vector; error bars: standard deviation.

D Vascular p-EphrinB in B16F10 tumors from mice transduced with control (pcDNA) and TNYL-RAW expression vectors.

Scale bars (A= 50μm; B= 200μm; D= 50μm).

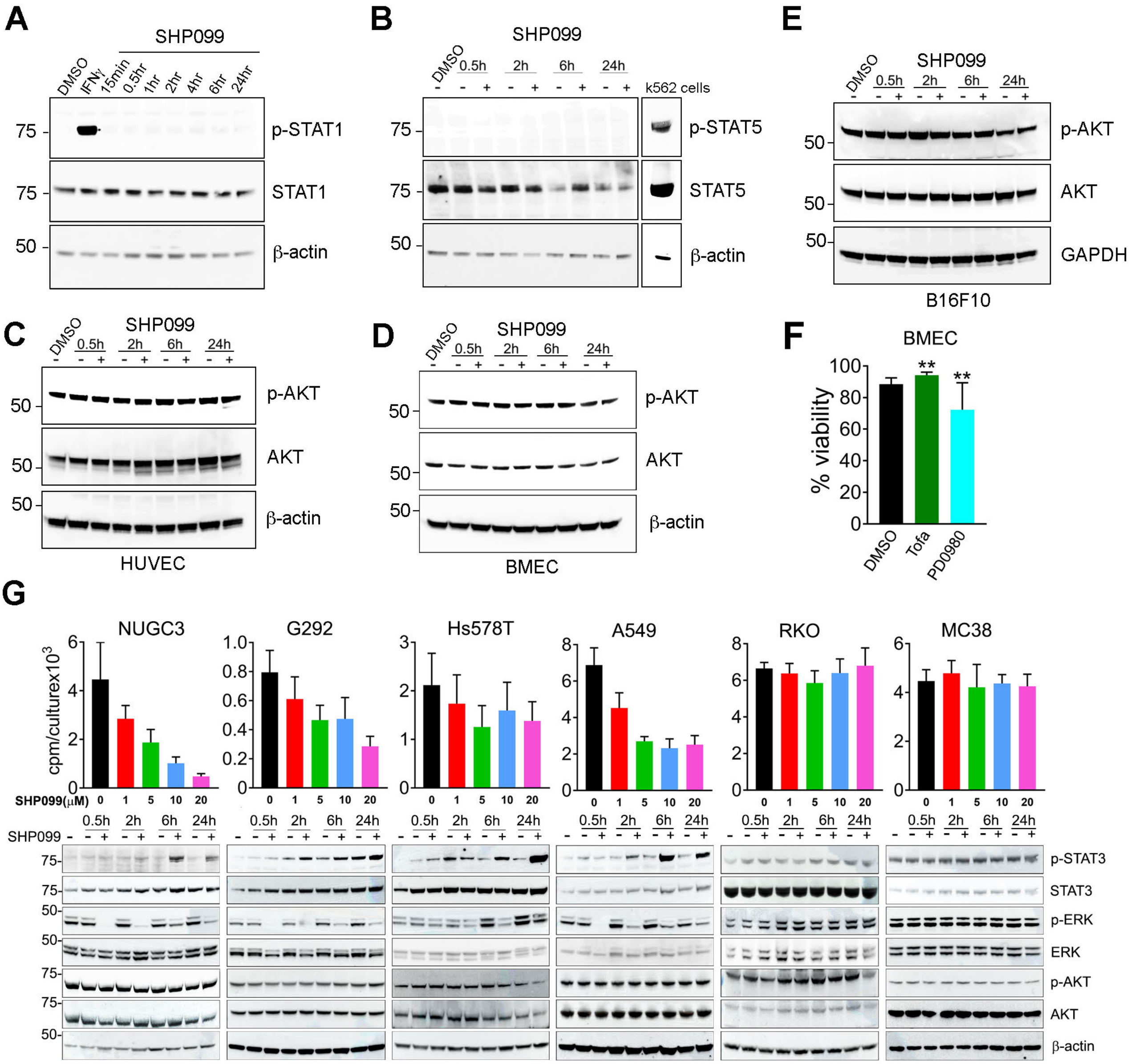

Appendix Figure S2

A-D SHP099 does not activate STAT1 (Tyr701) (A), STAT5 (Tyr694) (B) or AKT (Ser437) (C, D) in endothelial cells. BMEC and HUVEC were incubated in medium only or with SHP099 (A,C: HUVEC 5µM; B,D: BMEC 20µM); immunoblotting results. K562 cells used as positive control for p-STAT5.

E SHP099 (20µM) minimally changes AKT activity in B16F10 by immunoblotting.

F Tofacitinib (Tofa, 50nM) enhances and PD098059 (PD0980, 10mM) reduces BMEC viability after 72-hour culture; the results from triplicate cultures (expressed as mean % viability compared to control) are representative of 3 experiments.
**P<0.01; two-tailed Student's t-test; error bars: S.D.

G Effects of SHP099 on the proliferation and activity of STAT3 and AKT in cancer cell lines by immunoblotting; error bars: SD, 5 replicates; representative of 3 experiments.

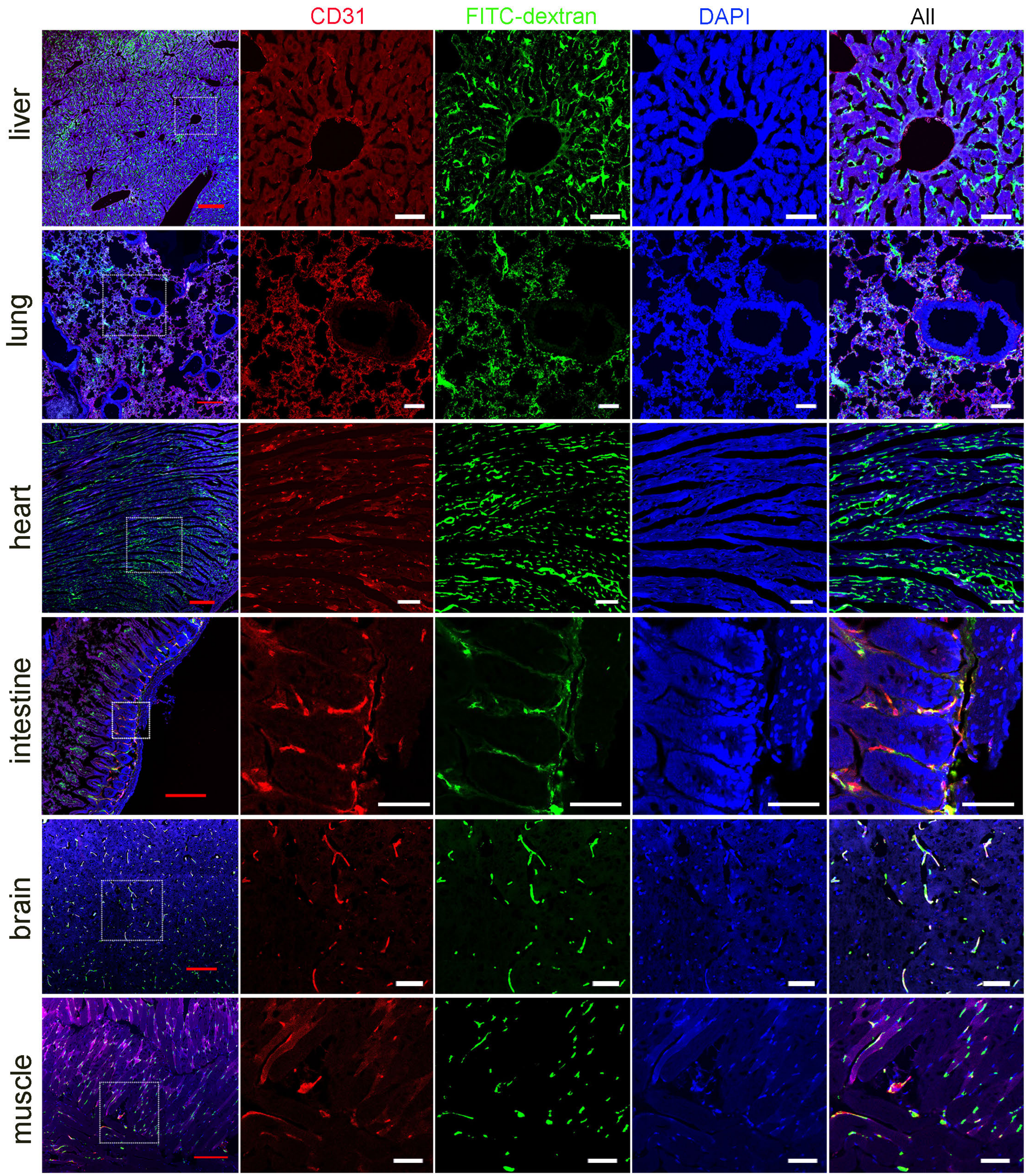

Appendix Figure S3 Legend in the next page

Appendix Figure S3

Blood vessels and blood perfusion in tissues from B16F10 tumor-bearing mice treated with SHP099 (100 mg/kg/day; 12 days). Blood vessels are identified by CD31 immunostaining (red); blood perfusion is visualized by FITC-dextran (2.000.000 mol weight, green); cell nuclei are visualized by DAPI (blue). Immunofluorescence confocal imaging; insets in the left panels are magnified in the adjacent panels; red scale bars: 200 µm; white scale bars: 50µm.

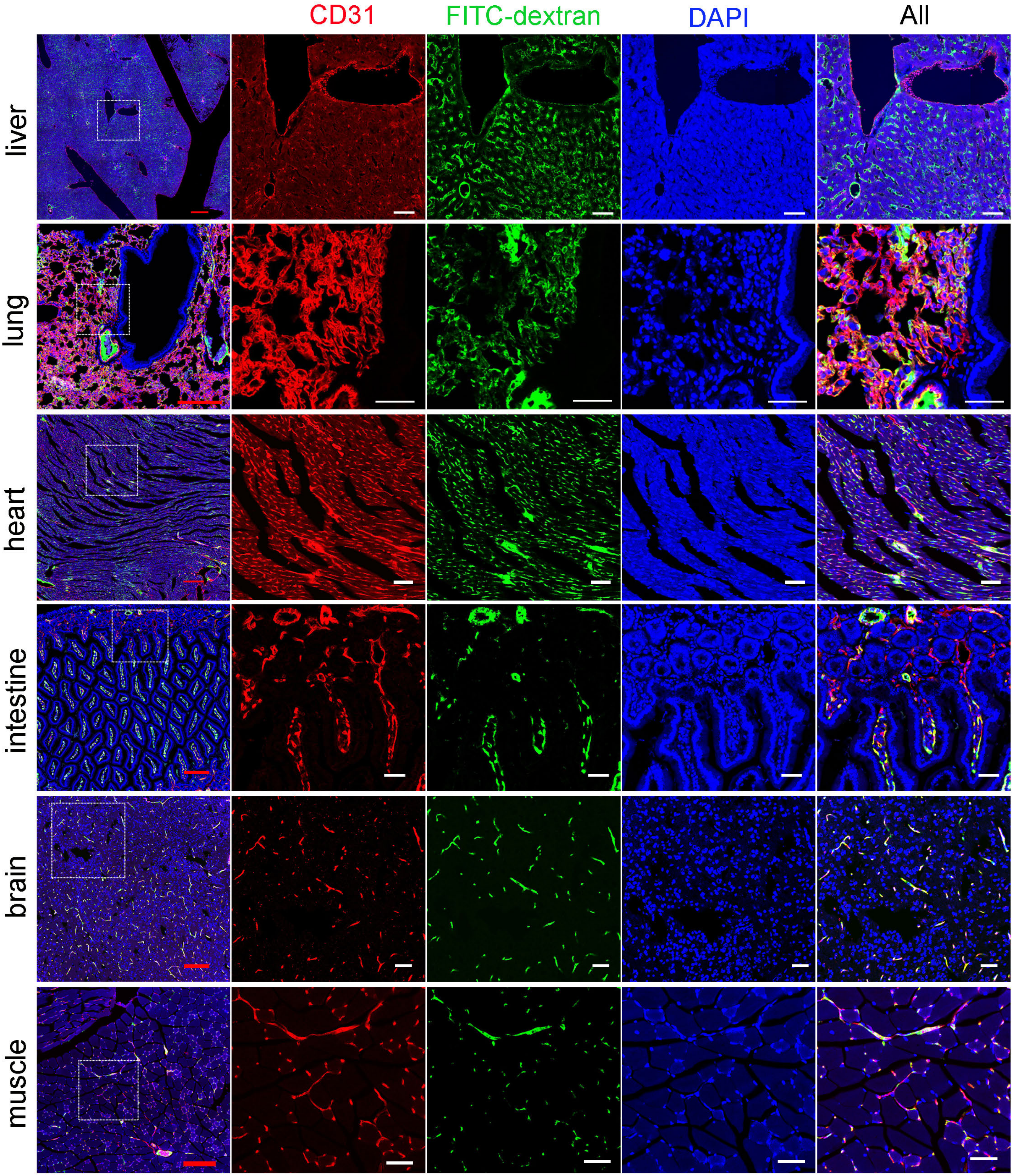

Appendix Figure S4 Legend in the next page

Appendix Figure S4

Vasculature of the indicated tissues from mice treated with SHP099+AMG386 by FITC-dextran perfusion and CD31 immunostaining; scale bar: red: 200µm; white: 50µm.

Appendix Table S1

List of p-values

**Figure 2**	
**panel B**	*p* value
melanoma	
ANGPT2	0.0064
colon carcinoma	
ANGPT2	1.76E-17
ANGPT1	2.80E-11
PDGFA	4.81E-06
FGF2	3.60E-11
VEGFA	6.11E-21
**panel I**	*p* value
HUVEC	9.12106E-06
BMEC	0.000421333
HDMEC	7.27297E-06

**Figure 3**	
**panel C**	*p* value
HUVEC	
DMSO vs. SHP099	7.52489E-12
DMSO vs. Tofa	0.005131187
BMEC	
DMSO vs. SHP099	0.0149
DMSO vs. Tofa	0.0135
**panel E**	*p* value
HUVEC	
DMSO vs. PD0980	0.0258
DMSO vs. SHP099	2.07372E-06
BMEC	
DMSO vs. PD0980	0.0208
DMSO vs. SHP099	0.001016572

**Figure 4**					
**panel A**	*p* value	**panel B**	*p* value	**panel C**	*p* value
tumor volumes		tumor volumes		tumor volumes	
day 10	0.031698158	*day 11*	0.041635486	*day 9*	0.029569904
day 11	0.01064093	*day 12*	0.001901178	*day 10*	0.019653525
day 12	0.001736328				
tumor weight		tumor weight	0.0104	tumor weight	0.0376
control vs. SHP099	0.025				
**panel F**	*p* value	**panel G**	*p* value	**panel I**	*p* value
control vs. SHP099	0.015	*control vs. SHP099*	0.0123	*control vs. SHP099*	1.69784E-24
**panel K**	*p* value	**panel L**	*p* value	**panel N**	*p* value
control vs. SHP099	0.0154	*control vs. SHP099*	0.0139	*control vs. SHP099*	0.0253

Figure 5

**panel A**	*p* value
tumor volumes	
day14	0.041372
day15	0.048132
day16	0.032080114
day17	0.007884946
tumor weight	0.019490968
**panel C**	*p* value
control vs SHP099	0.0252
**panel E**	
p-LKO-control vs	p value
p-LKO-SHP099	0.000382231
shSHP2-control vs	*p* value
shSHP2-SHP099	0.000696881
**panel H**	*p* value
control vs SHP099	0.0311
**panel J**	*p* value
control vs SHP099	0.0034
**panel K**	*p* value
control vs SHP099	2.46465E-05
**panel L**	*p* value
control vs SHP099	0.000036
**panel M**	*p* value
control vs SHP099	1.43391E-10

Figure 6

**panel A**	p value	**panel I**	p value
tumor weight		control vs. SHP099	0.001503528
control vs SHP099	0.002182408	control vs. AMG386	0.000698788
control vs AMG386	0.002026037	control vs. combined	<1.000000000000000e-004
control vs combined	<1.000000000000000e-004		
		**panel J**	p value
**panel B**	p value	control vs. SHP099	0.001759061
tumor weight		control vs. AMG386	0.006431131
control vs SHP099	0.00181627	control vs. combined	<1.000000000000000e-004
control vs AMG386	0.006171783		
control vs combined	<1.000000000000000e-004		
**panel D**	p value	**panel K**	p value
control vs AMG386	3.0849E-146	control vs. SHP099	0.028262091
		control vs. AMG386	0.049101428
**panel F**	p value	control vs. combined	<1.000000000000000e-004
control vs AMG386	6.57895E-13		
**panel H**	p value	**panel L**	
control vs. SHP099	<1.000000000000000e-004	control vs. SHP099	0.0015
control vs. AMG386	0.005645461	control vs. AMG386	0.001
control vs. combined	<1.000000000000000e-004	control vs. combined	0.0009

Figure 7

**panel B**	p value	**panel F**	p value
control vs. SHP099	<1.000000000000000e-004	*control vs. AMG386*	<1.000000000000000e-004
control vs. AMG386	0.00024779	*control vs. combined*	<1.000000000000000e-004
control vs. combined	0.001804829		
**panel D**	p value		
control vs. SHP099	<1.000000000000000e-004		
control vs. AMG386	0.002041293		
control vs. combined	<1.000000000000000e-004		

Figure 8

panel A	*p* value
control vs. SHP099	0.000421324
control vs. AMG386	0.023959973
control vs. combined	<1.000000000000000e-004

panel D	*p* value
control vs. SHP099	0.014913321
control vs. AMG386	0.025943219
control vs. combined	0.000231581

panel E	*p* value
control vs. SHP099	<1.000000000000000e-004
control vs. AMG386	<1.000000000000000e-004
control vs. combined	<1.000000000000000e-004

panel F	*p* value
control vs. SHP099	0.0026
control vs. AMG386	0.0057
control vs. combined	0.0003

panel G	*p* value
control vs. SHP099	<1.000000000000000e-004
control vs. AMG386	<1.000000000000000e-004
control vs. combined	<1.000000000000000e-004

panel I	*p* value
control vs. SHP099	0.018239072
control vs. AMG386	0.011129898
control vs. combined	0.000101669

panel J	*p* value
control vs. SHP099	<1.000000000000000e-004
control vs. AMG386	0.007171034
control vs. combined	0.023349744

panel K	*p* value
control vs. SHP099	<1.000000000000000e-004
control vs. AMG386	<1.000000000000000e-004
control vs. combined	<1.000000000000000e-004

panel L	*p* value
control vs. AMG386	<1.000000000000000e-004
control vs. combined	<1.000000000000000e-004

Figure EV1

panel E	*p* value
control vs. SHP099	0.015178

Figure EV3

panel F	*p* value
control vs. SHP099	0

panel G	p value
control vs. SHP099	0.96

Figure EV4

panel B	*p* value
control vs. SHP099	0.7306

Appendix Figure S2

panel F	*p* value
DMSO vs. Tofa	0.001615216
DMSO vs. PD0980	0.006125818

Figure EV2

panel A	*p* value
BMEC	
24h	1.65E-08
48h	1.78804E-07
72h	7.65253E-10
HUVEC	
24h	0.63735
48h	1.19115E-06
72h	7.82946E-13

Figure EV5

panel E	*p* value
p-Tie2	8.8245E-145
p-EphrinB	1.77622E-46

panel G	*p* value
control vs. SHP099	0.136

panel H	*p* value
control vs. SHP099	0.4681
