# Supplementary material for: Targeting the SHP2 phosphatase promotes vascular damage and inhibition of tumor growth
Source: EMBO Mol Med. 2021 Jun 8;13(7):e14089. doi: 10.15252/emmm.202114089 (PMC8261520; doi:10.15252/emmm.202114089)
Supplement: Supplementary file 2 — Expanded View Figures PDF [file EMMM-13-e14089-s007.pdf]

Expanded View Figures

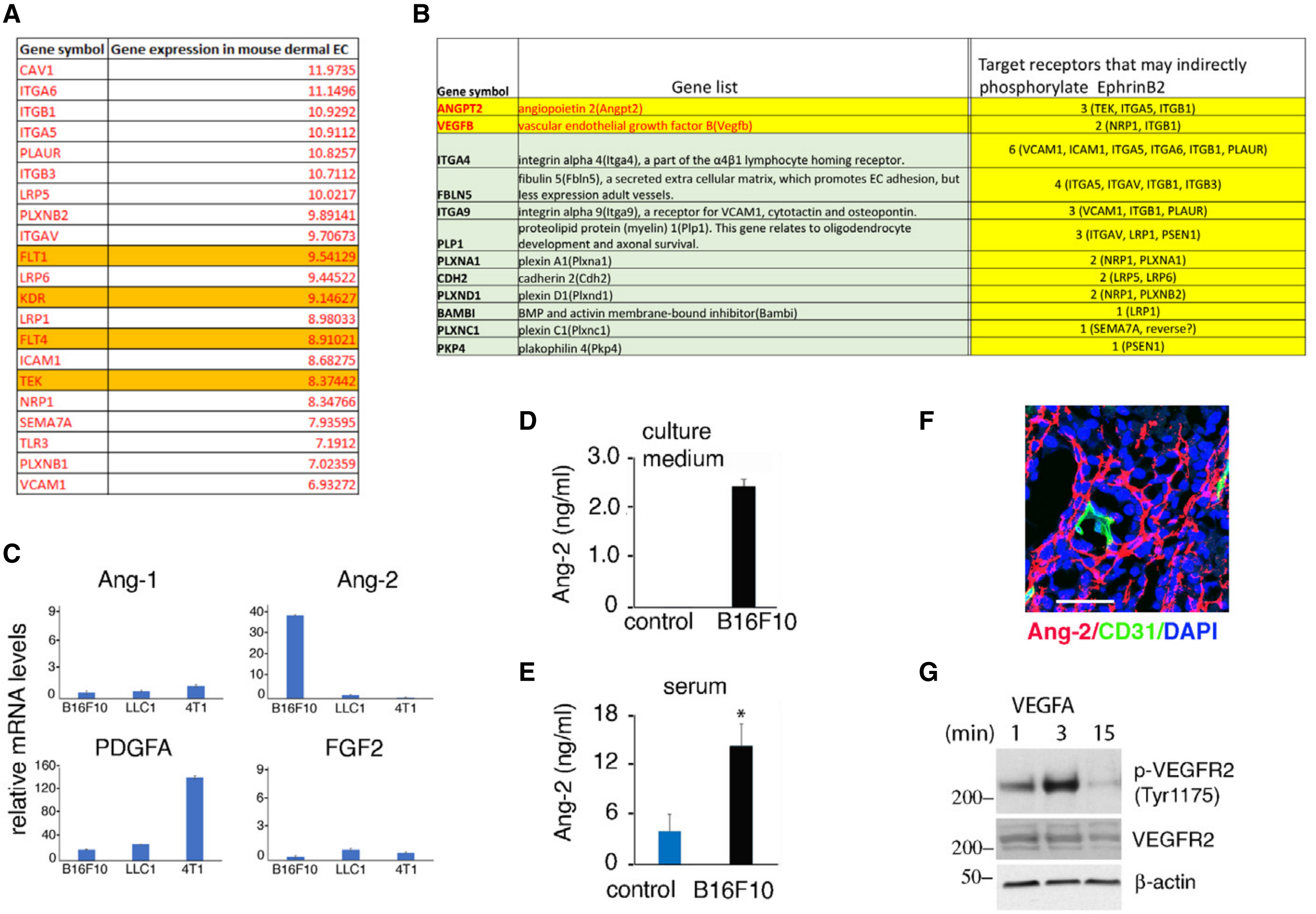

A

Gene symbol	Gene expression in mouse dermal EC
CAV1	11.9735
ITGA6	11.1496
ITGB1	10.9292
ITGA5	10.9112
PLAUR	10.8257
ITGB3	10.7112
LRP5	10.0217
PLXNB2	9.89141
ITGAV	9.70673
FLT1	9.54129
LRP6	9.44522
KDR	9.14627
LRP1	8.98033
FLT4	8.91021
ICAM1	8.68275
TEK	8.37442
NRP1	8.34766
SEMA7A	7.93595
TLR3	7.1912
PLXNB1	7.02359
VCAM1	6.93272

B

Gene symbol	Gene list	Target receptors that may indirectly phosphorylate EphrinB2
ANGPT2	angiopoietin 2(Angpt2)	3 (TEK, ITGA5, ITGB1)
VEGFB	vascular endothelial growth factor B(Vegfb)	2 (NRP1, ITGB1)
ITGA4	integrin alpha 4(Itga4), a part of the α4β1 lymphocyte homing receptor.	6 (VCAM1, ICAM1, ITGA5, ITGA6, ITGB1, PLAUR)
FBLN5	fibulin 5(Fbln5), a secreted extra cellular matrix, which promotes EC adhesion, but less expression adult vessels.	4 (ITGA5, ITGAV, ITGB1, ITGB3)
ITGA9	integrin alpha 9(Itga9), a receptor for VCAM1, cytotactin and osteopontin.	3 (VCAM1, ITGB1, PLAUR)
PLP1	proteolipid protein (myelin) 1(Plp1). This gene relates to oligodendrocyte development and axonal survival.	3 (ITGAV, LRP1, PSEN1)
PLXNA1	plexin A1(Plxna1)	2 (NRP1, PLXNA1)
CDH2	cadherin 2(Cdh2)	2 (LRP5, LRP6)
PLXND1	plexin D1(Plxnd1)	2 (NRP1, PLXNB2)
BAMBI	BMP and activin membrane-bound inhibitor(Bambi)	1 (LRP1)
PLXNC1	plexin C1(Plxnc1)	1 (SEMA7A, reverse?)
PKP4	plakophilin 4(Pkp4)	1 (PSEN1)

Figure EV1. B16F10-derived activators of endothelial EphrinB.

A Receptors kinase/kinase genes expressed in murine dermal microvascular endothelial cells (EC) with the potential to phosphorylate EphrinB2.

B Soluble factors/transmembrane protein genes that have the potential to phosphorylate EphrinB2 and are expressed at significantly higher levels in B16F10 cells compared to LLC1 and 4T1.

C Relative expression levels of Ang-1, Ang-2, PDGFA, and FGF2 in B16F10, LLC1, and 4T1 tumors from mice. Error bars: ± SD; triplicate measurements, three independent experiments.

D Ang2 levels in culture supernatants of B16F10 cells and medium only (control). Error bars: ± SD; triplicate measurements, three independent experiments.

E Ang2 levels in serum of control mice ($n = 4$) and mice bearing B16F10 tumors ($n = 3$). Error bars: ± SD; triplicate measurements. *P* values from two-tailed Student's *t*-test; $^*P < 0.05$.

F Ang2 levels in B16F10 tumor tissue (scale bar: 50 μm).

G VEGF-A (100 ng/ml) activates VEGFR2 in HUVEC; immunoblotting.

 © 2021 The Authors

Figure EV2. Effects of SHP099 on endothelial and B16F10 cells.

A SHP099 reduces endothelial cell proliferation; relative means (SD: error bars) of triplicate cultures; representative of three experiments. *P* values from two-tailed Student's *t*-test; $^{***}P < 0.001$.
B Representative cell cycle flow cytometry profiles of endothelial cells with or without SHP099.
C Representative cell viability flow cytometry profiles of endothelial cells with or without SHP099.
D Cell growth curves after rescue of viable cells from control and SHP099-supplemented cultures for 72 h; IncuCyte live-cell imaging; % mean confluency (SD), triplicate cultures; upper panel: BMEC; lower panel: HUVEC.
E Effect of Matrigel on B16F10 cell viability; representative of three experiments; five replicate cultures.
F Death-related proteins in HUVEC after 72-h culture in control or SPH099-supplemented medium; results: fold change from control.
G SHP099 reduces VE-cadherin in BMEC and HUVEC; 72 h incubation.
H SHP099 increases JNK3 in BMEC and HUVEC. SHP099 concentration, all experiments: HUVEC and HDMAC 5 mM; BMEC 20 mM.

Data information: Data are presented as mean ± SD.

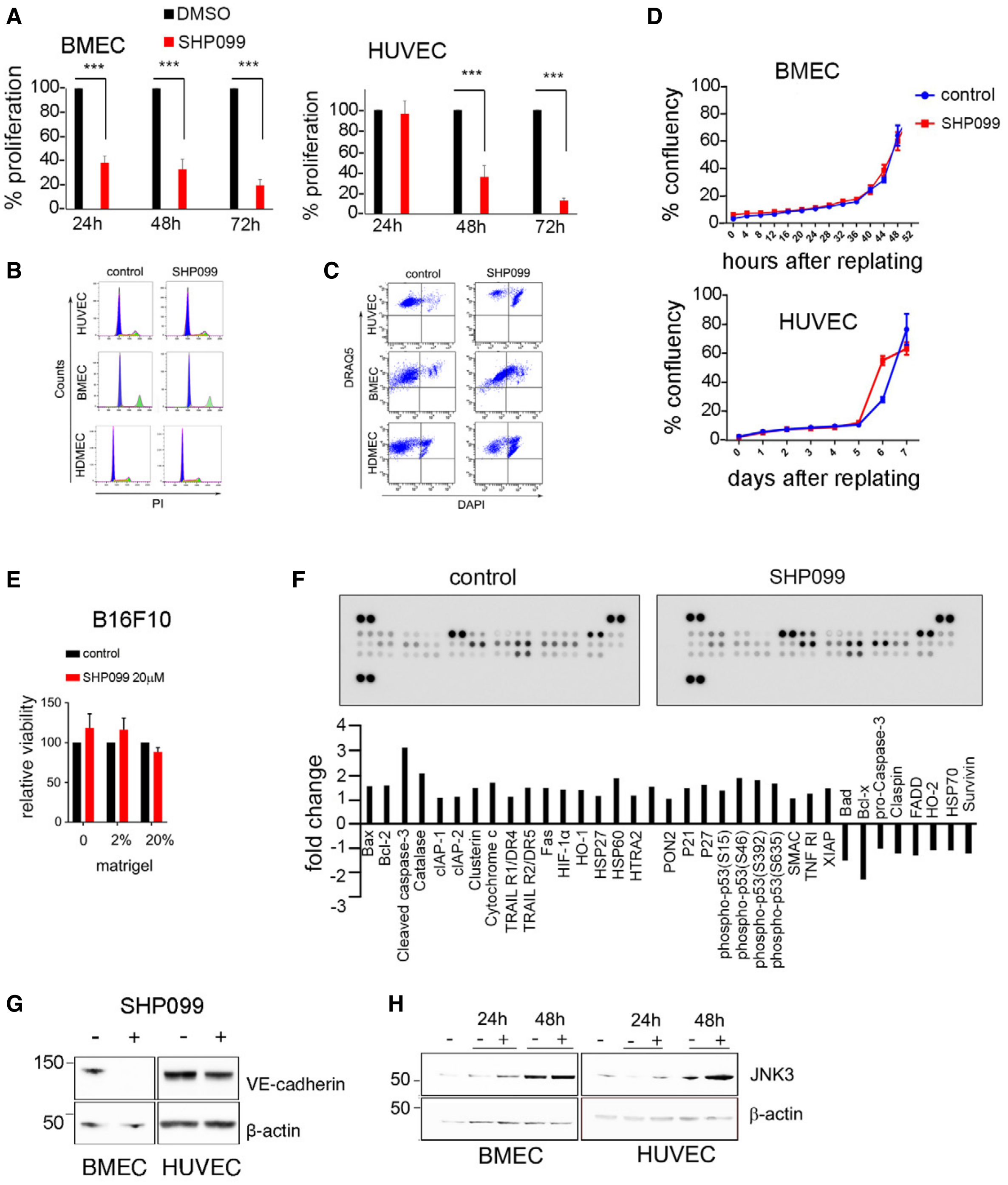

Figure EV2.

© 2021 The Authors

Figure EV3. SHP099 treatment of B16F10 tumor-bearing mice.

A Mouse body weight after treatment with SHP099 (100 mg/kg). The number of mice/group (mouse no.)/experiment is indicated below each bar.

B Mouse body weight after treatment with SHP099 (100 mg/kg), AMG386 (5.6 mg/kg), or SHP099 (100 mg/kg) plus AMG386 (5.6 mg/kg). Error bars: SD. The number of mice/group (mouse no.)/experiment is indicated below each bar.

C Erythrocytes and FITC-dextran extravasation from the tumor vasculature of SHP099-treated mice. CD31 (red): tumor vessels; DAPI: cell nuclei. Arrows: auto-fluorescent erythrocytes; scale bar 10 μm.

D Segmental "vascular sleeve" in a representative tumor vessel; SHP099-treated mouse; scale bar 10 mm. Arrowheads point to a Collagen $IV^+/CD31^-$ vessel segment.

E Cleaved caspase-3 in tumor cells (yellow arrows) and vascular endothelial cells (white arrows) of control and SHP099-treated mice; scale bar 100 μm.

F Quantification of cleaved caspase-3^+ cells in control and SHP099-treated tumors (n = 3/group); number of cells counted: control (n = 1,776), SHP099 (n = 3,252).

G F4/80^+ cells visualized in control and SHP099-treated tumors are quantified in the bar graph (n = 3/group); Error bars: ± SD; scale bar 50 μm.

Data information: P values from two-tailed Student's t-test; ***$P < 0.001$; NS: not significant.

© 2021 The Authors

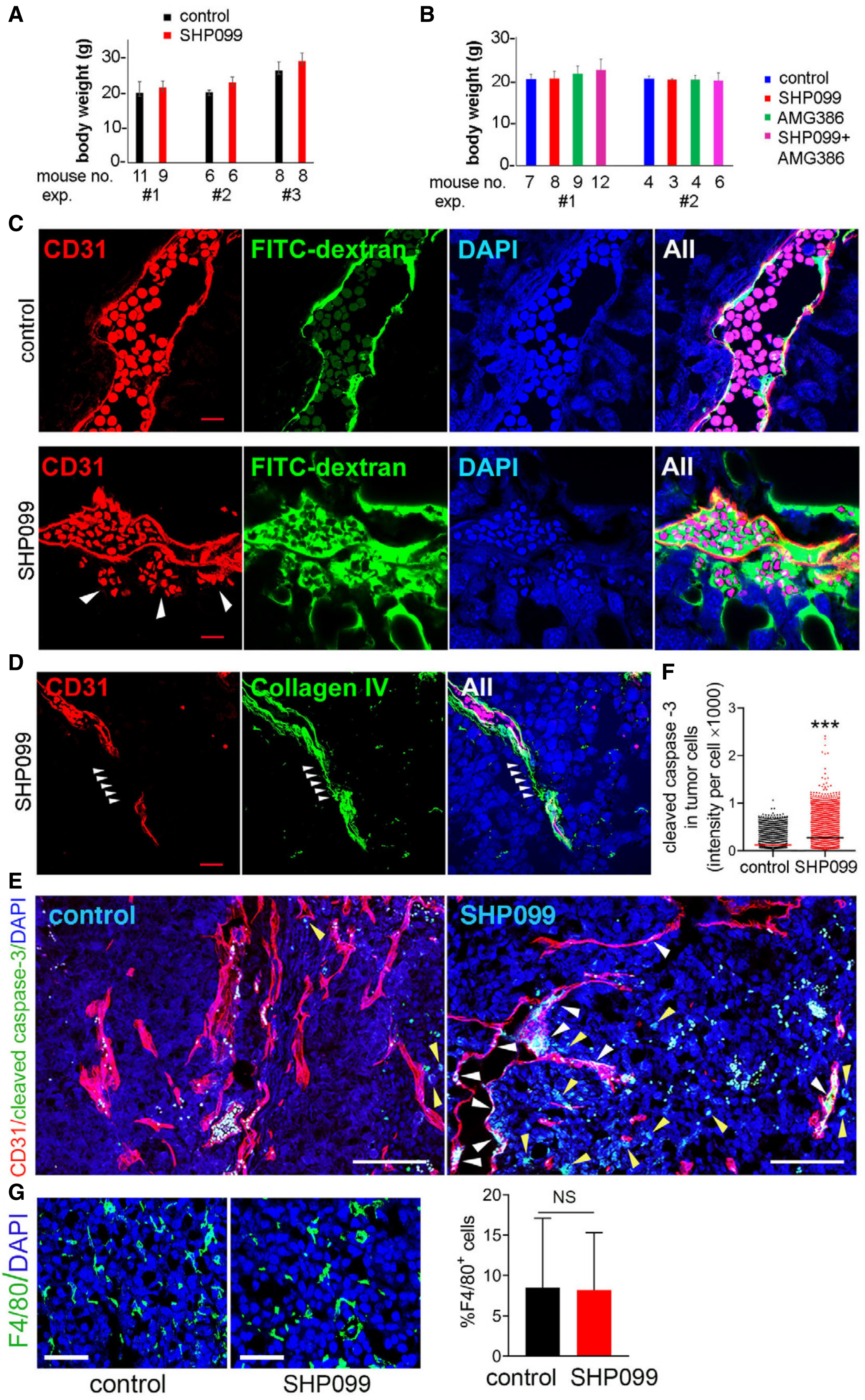

Figure EV3.

 © 2021 The Authors

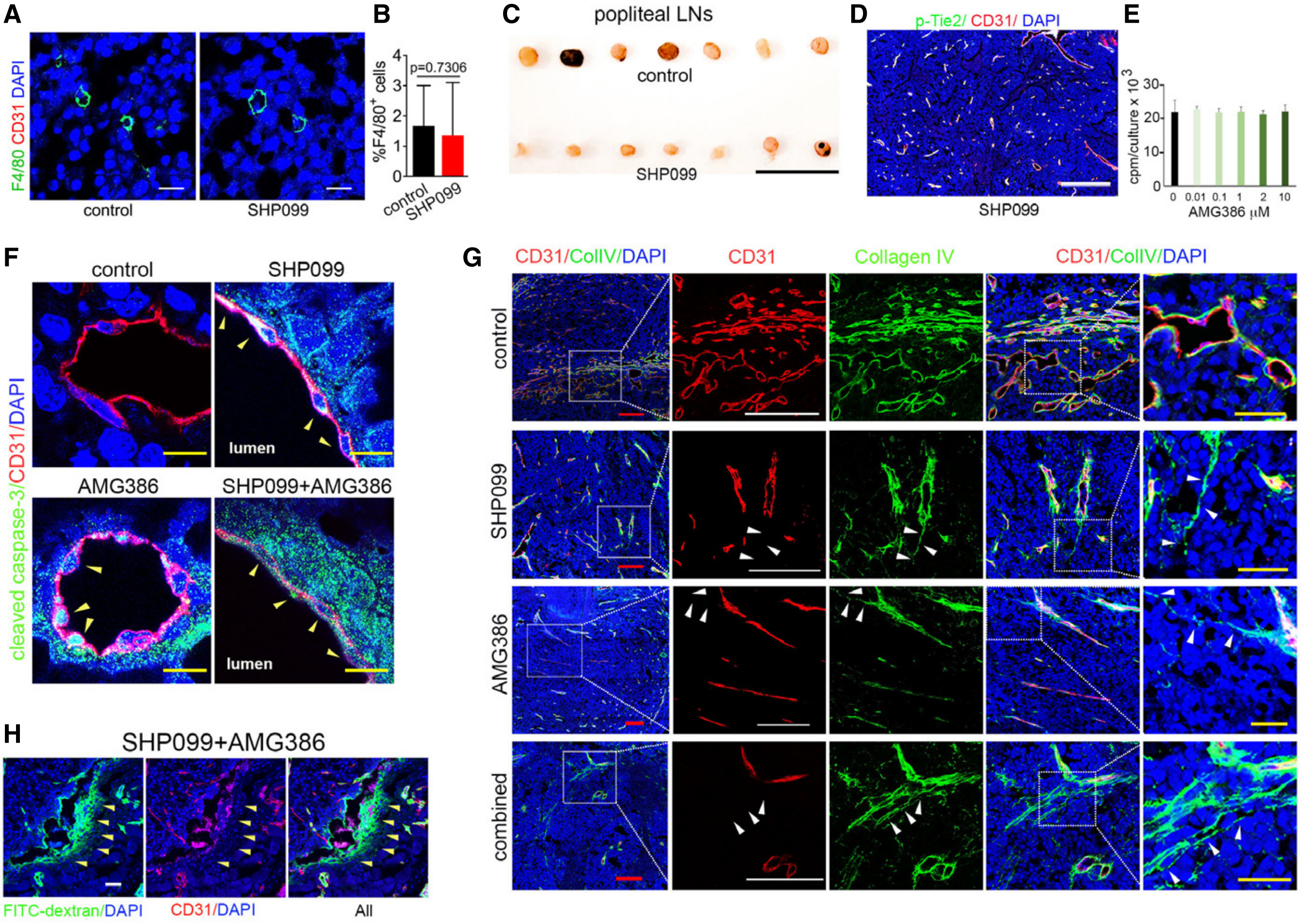

Figure EV4. Effects of SHP099 on B16F10 microenvironment and lymph node dissemination.

A F4/80 macrophages in B16F10 tumor from NSG mice; scale bar = 50 µm.
B Quantification of F4/80 macrophages shown in A. Error bars: ± SD.
C Representative popliteal lymph nodes (LN) from control and SHP099-treated mice; scale bar = 1 cm.
D TIE2 is active in the tumor vasculature after SHP099 treatment; scale bar = 500 µm.
E AMG386 minimally affects B16F10 cell proliferation. Error bars: ± SD.
F Cleaved caspase-3 in the vasculature of treated B16F10 tumors; scale bars = 10 µm.
G Vascular sleeves in the vasculature of treated B16F10 tumors; scale bars = 200 µm.
H FITC-dextran extravasation in B16F10 tumors treated with SHP099 and AMG386; scale bar = 50 µm. (please note: scale bar was lost in the conversion from TIFF to PDF; we have substituted the PDF with a new one with the scale bar).

© 2021 The Authors

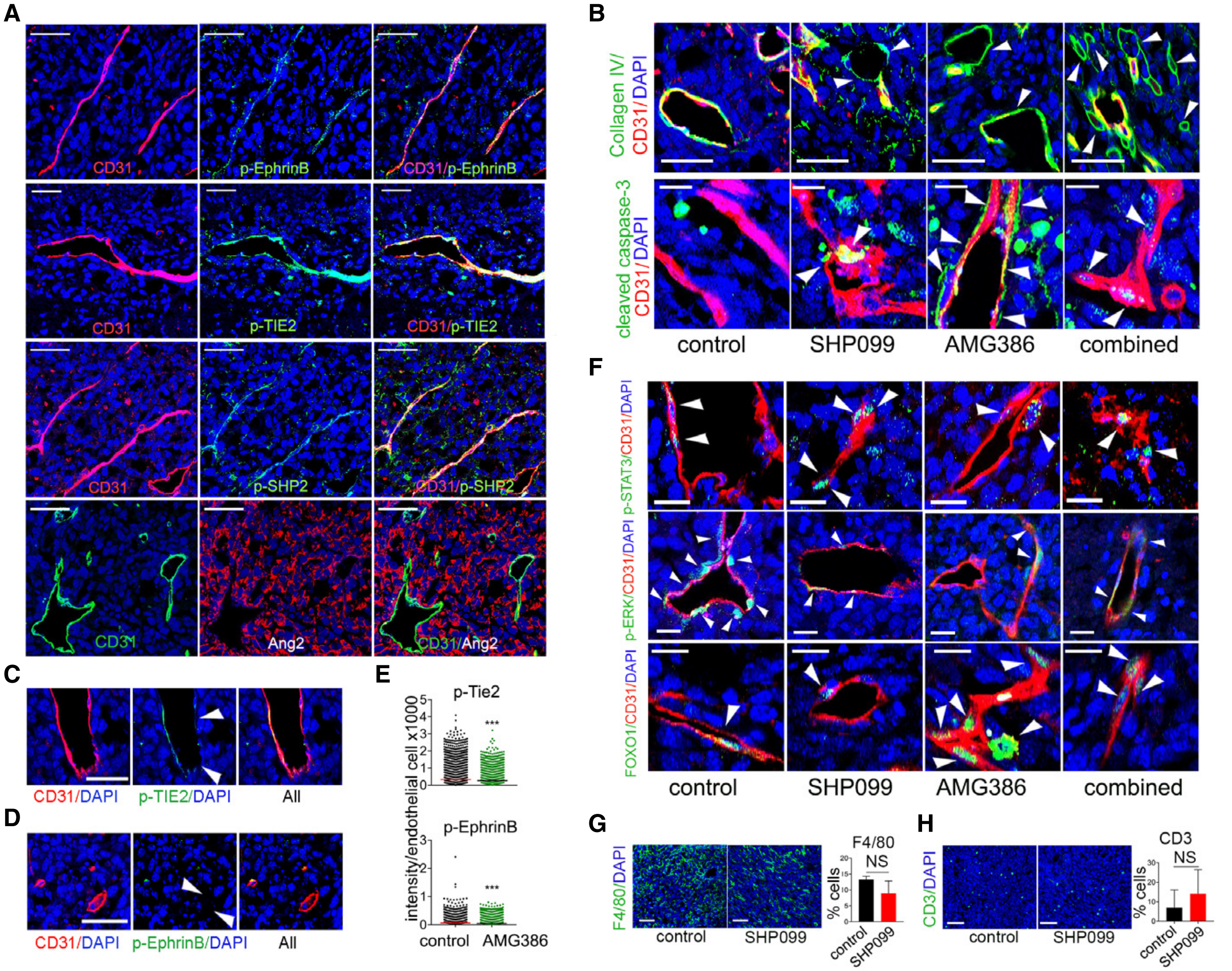

Figure EV5. Effects of SHP099 on MC38 tumors.

A Immunostaining of untreated MC38 tumors for the indicated markers; scale bar 50 μm.

B Vascular sleeves and cleaved caspase-3 visualized in MC38 tumors treated with SHP099 (100 mg/kg), AMG386 (5.6 mg/kg) or SHP099 (100 mg/kg) + AMG386 (56 mg/kg) and controls; scale bar 50 μm.

C p-Tie2 in MC38 tumors treated with AMG386 (5.6 mg/kg); scale bars 50 μm. Arrowheads point to $CD31^+$/p-$TIE2^-$ endothelial cells.

D p-EphrinB in MC38 tumors treated with AMG386 (5.6 mg/kg); scale bars 50 μm. Arrowheads point to $CD31^+$/p-$EphrinB^-$ endothelial cells.

E Quantification of vascular p-$Tie2^+$ and p-$EphrinB^+$ endothelial cells in AMG386-treated and control mice; n = 3 tumors/group. Number of $CD31^+$ endothelial cells counted: top (p-Tie), control (n = 12,285), AMG386 (n = 13,046); bottom (p-EphrinB), control (n = 19,411), AMG386 (n = 22,550).

F p-STAT3, p-ERK and FOXO1 immunostaining of MC38 tumors treated with SHP099 (100 mg/kg), AMG386 (5.6 mg/kg), SHP099 (100 mg/kg) + AMG386 (56 mg/kg) and controls; scale bars 10 μm (p-STAT3) and 20 μm (p-Tie2 and FOXO1).

G F4/80 immunostaining of control and SHP099-treated tumors (n = 3/group); scale bars 50 μm.

H CD3 immunostaining of control and SHP099-treated tumors (n = 3/group); scale bars 50 μm.

Data information: Error bars: SD; P values by two-tailed Student's t-test. ***$P < 0.001$; NS: not significant.

 © 2021 The Authors
