# Supplementary figures and images for: Targeting the SHP2 phosphatase promotes vascular damage and inhibition of tumor growth
Source: EMBO Mol Med. 2021 Jun 8;13(7):e14089. doi: 10.15252/emmm.202114089 (PMC8261520; doi:10.15252/emmm.202114089)

Figure 1A

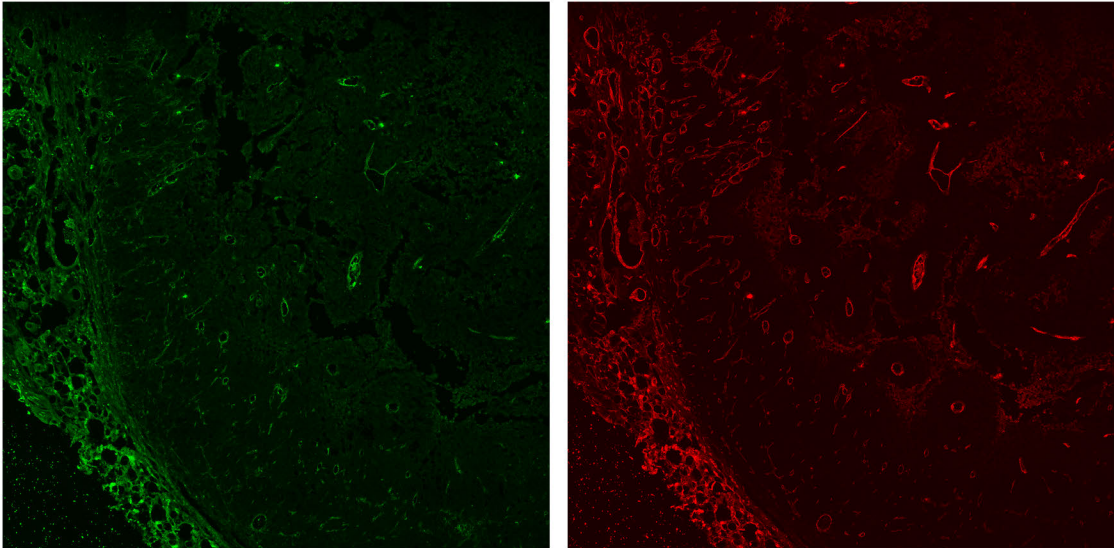

Figure 1C

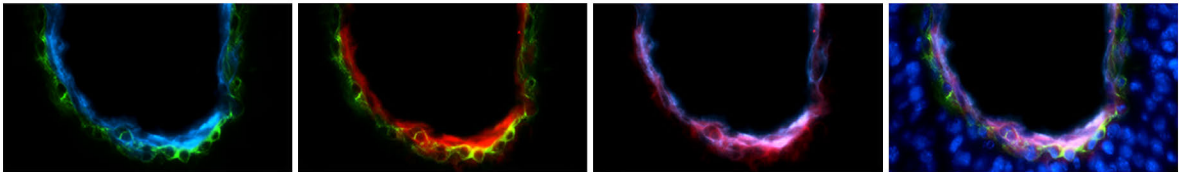

Figure 1D

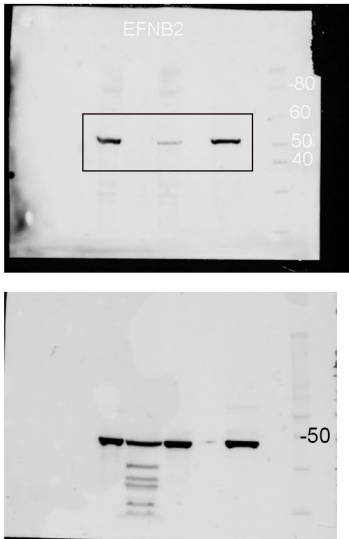

Figure 1E

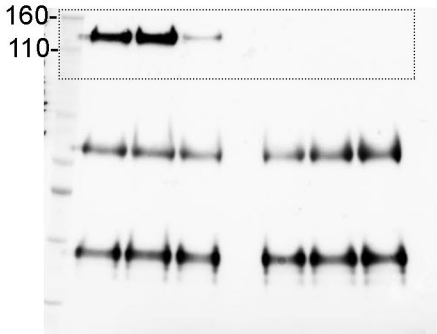

Figure 1F

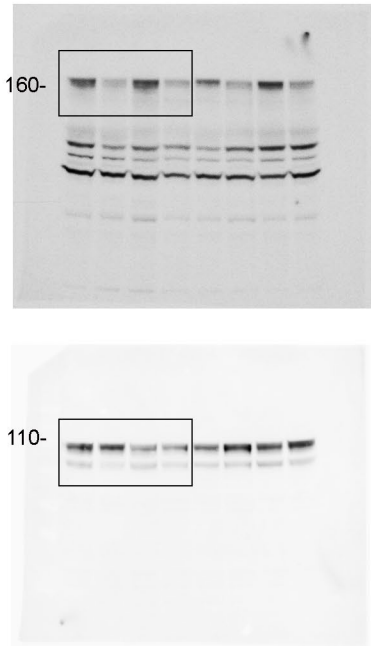

Figure 1H

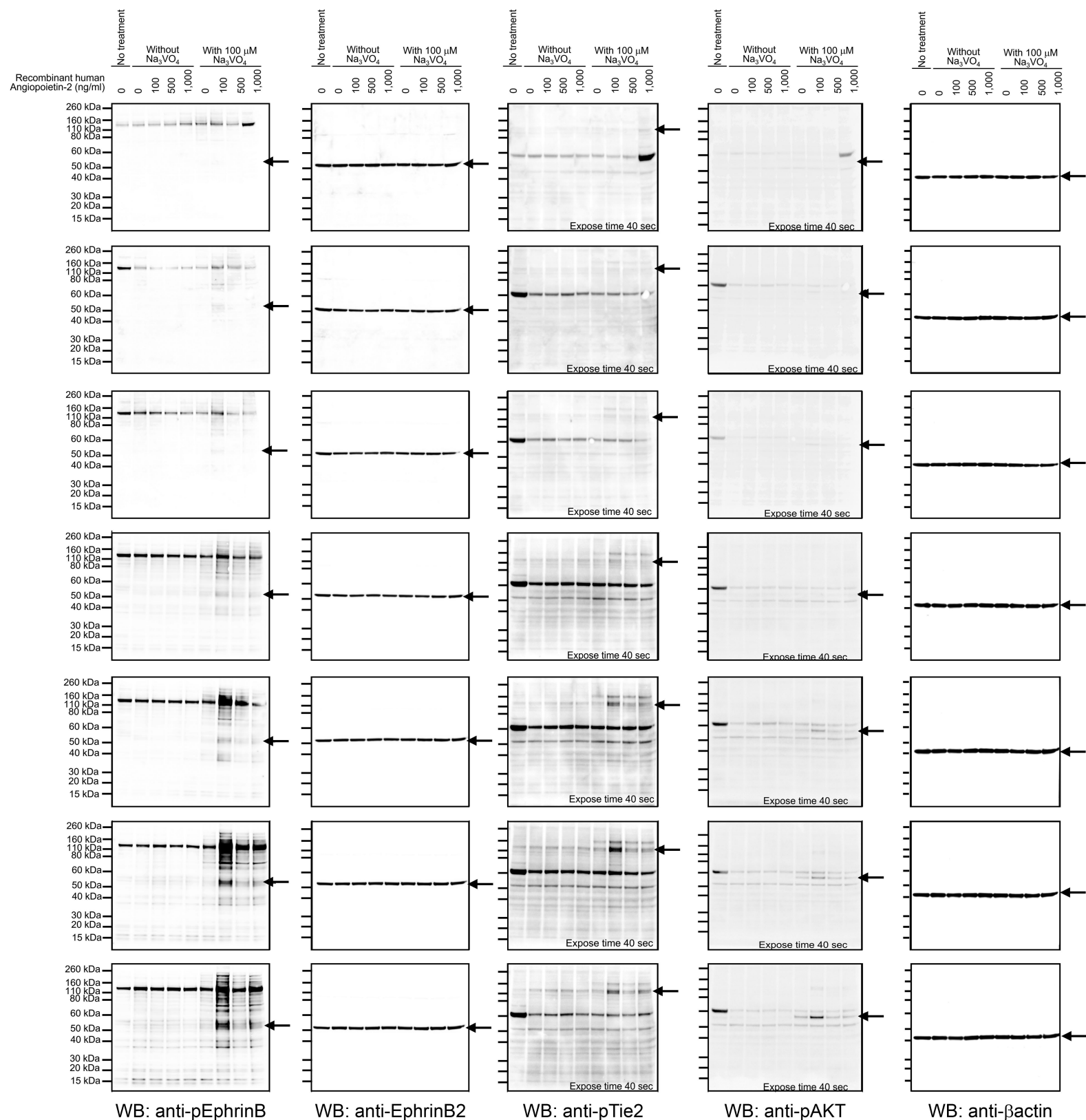

Supplement: Supplementary file 4 — Source Data for Figure 1 [file EMMM-13-e14089-s005.pdf]

Figure 2A

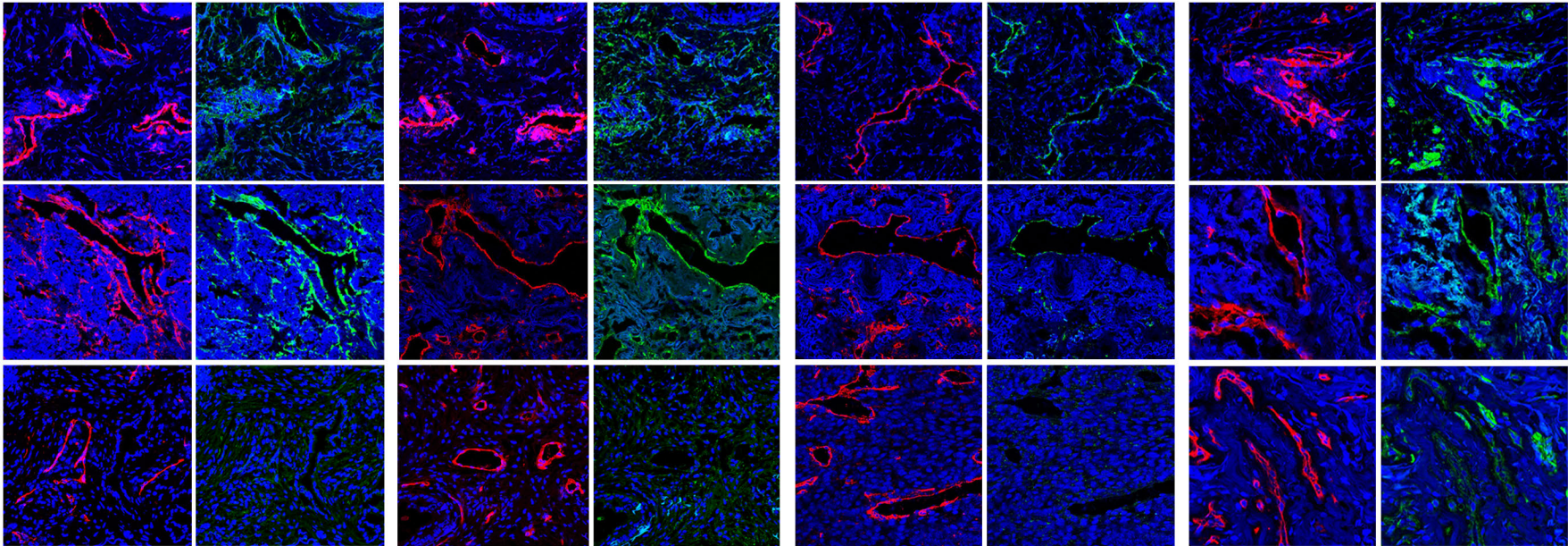

Figure 2F

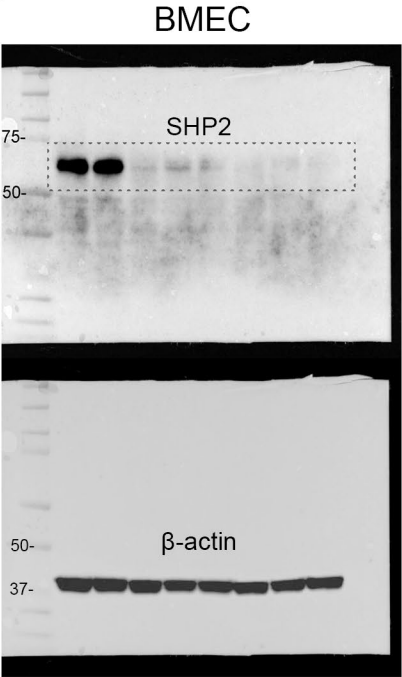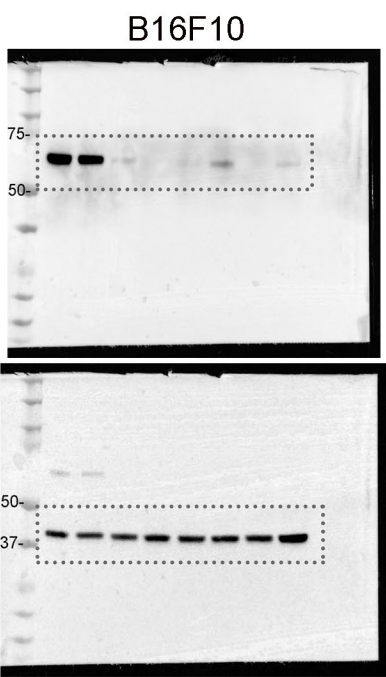

Supplement: Supplementary file 5 — Source Data for Figure 2 [file EMMM-13-e14089-s003.pdf]

Figure 3A

HUVEC

BMEC

B16F10

p-STAT3

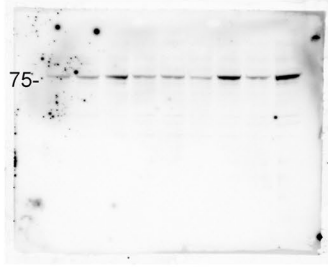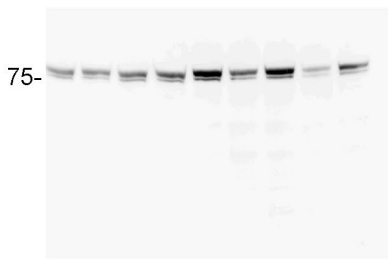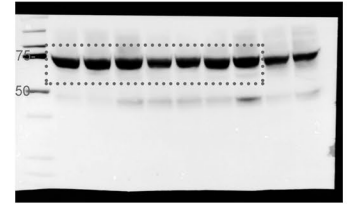

STAT3

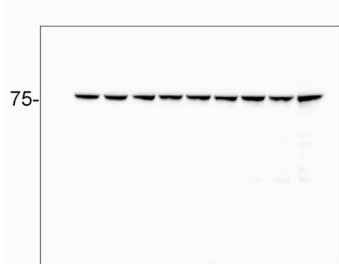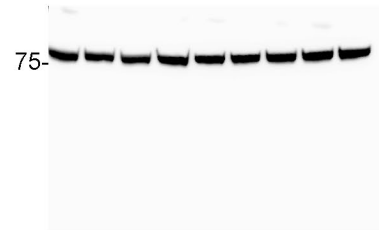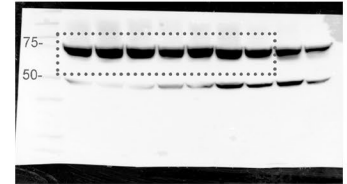

$\beta$ -actin

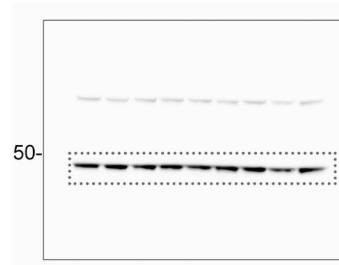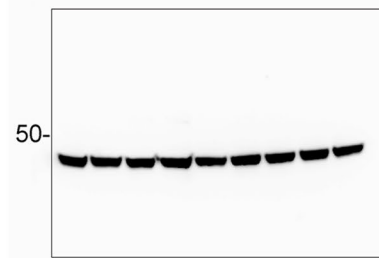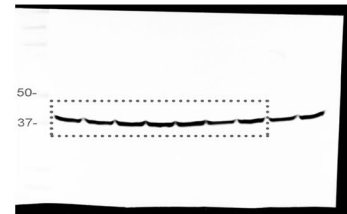

p-ERK

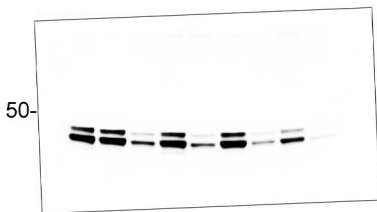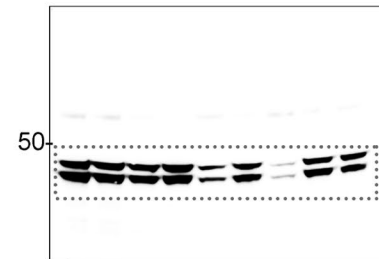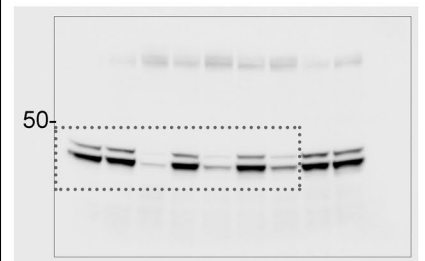

ERK

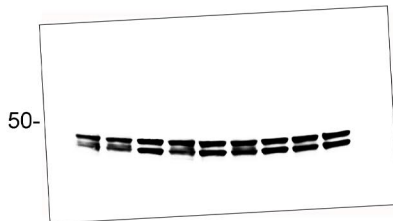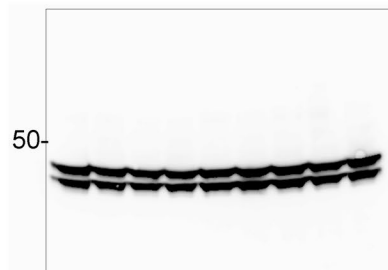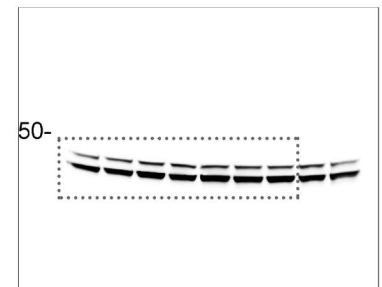

$\beta$ -actin

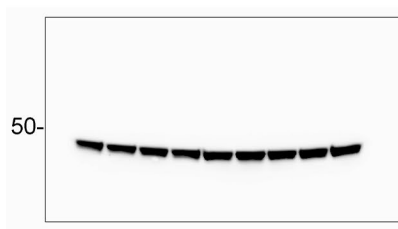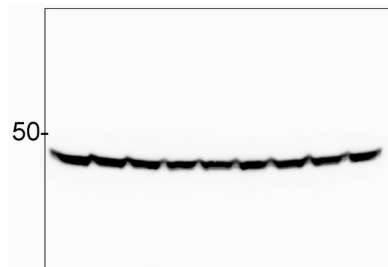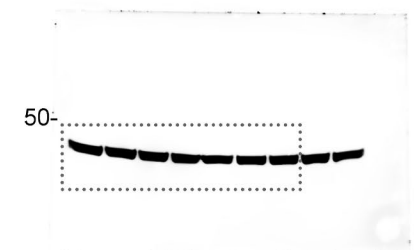

Figure 3D

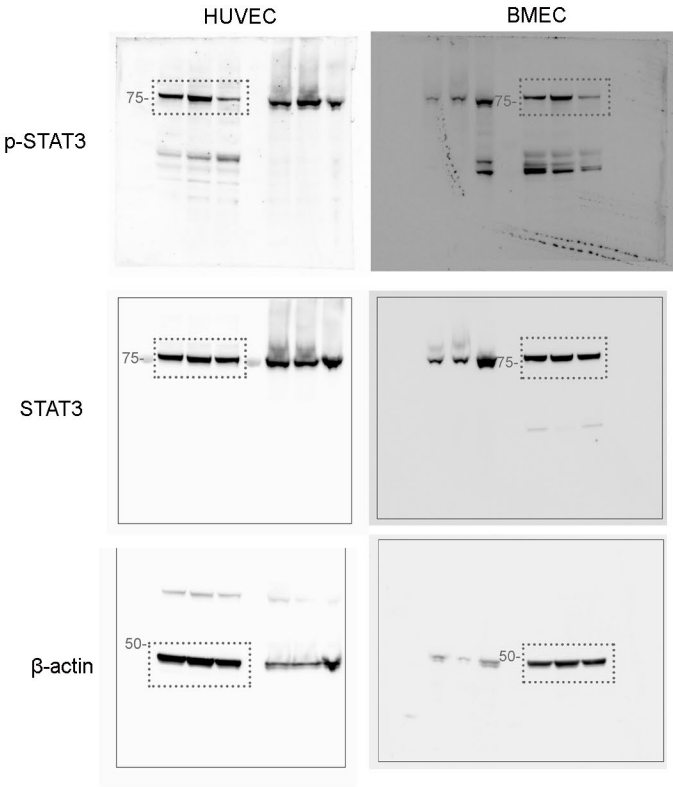

Figure 3F

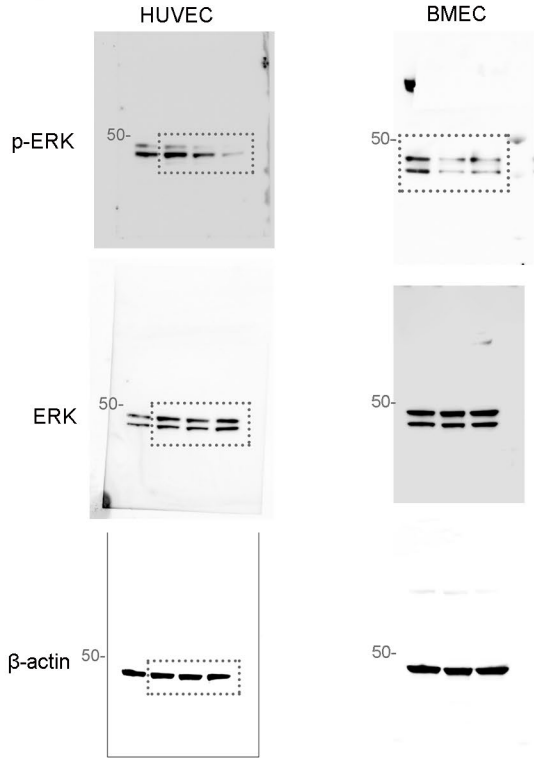

Figure 3H

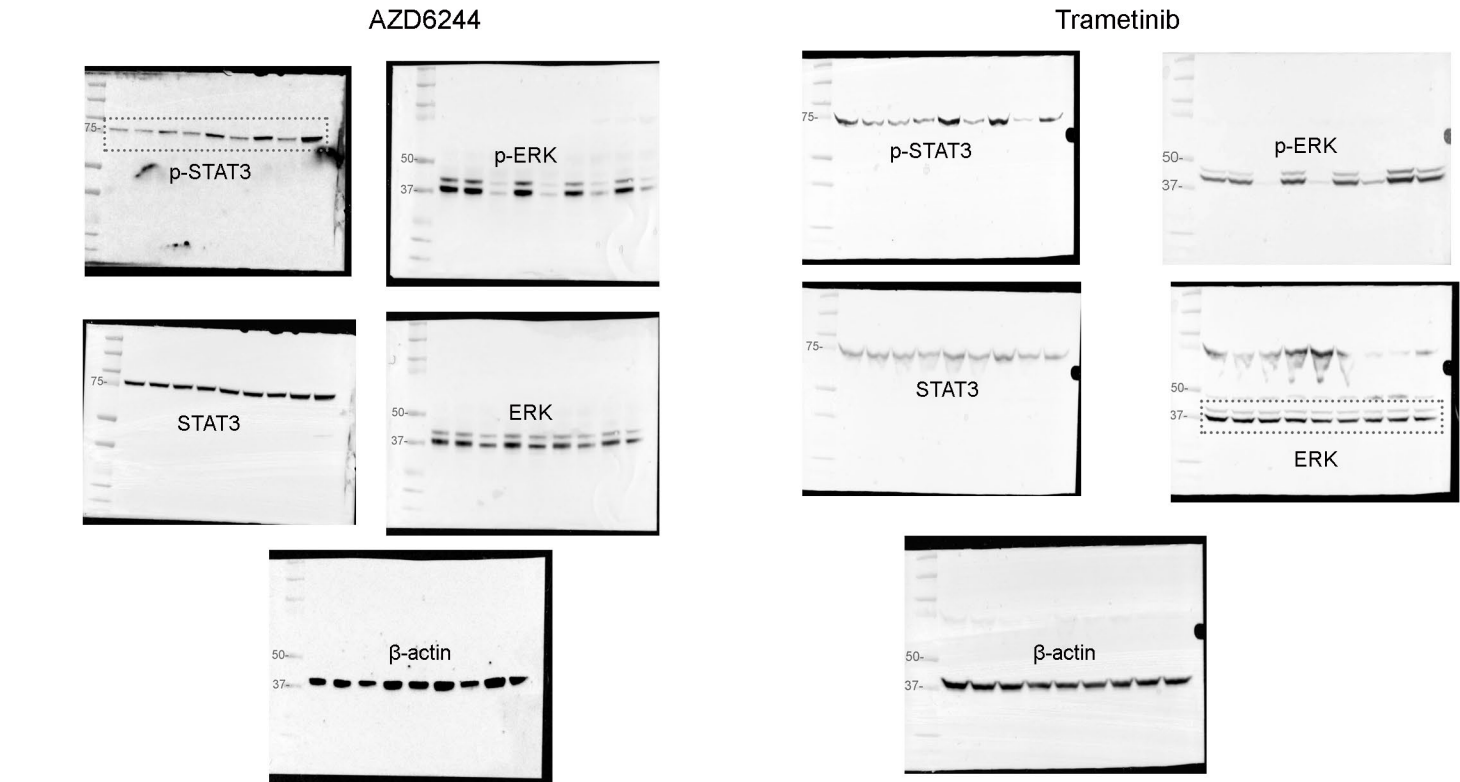

Supplement: Supplementary file 6 — Source Data for Figure 3 [file EMMM-13-e14089-s001.pdf]

Figure 6 C

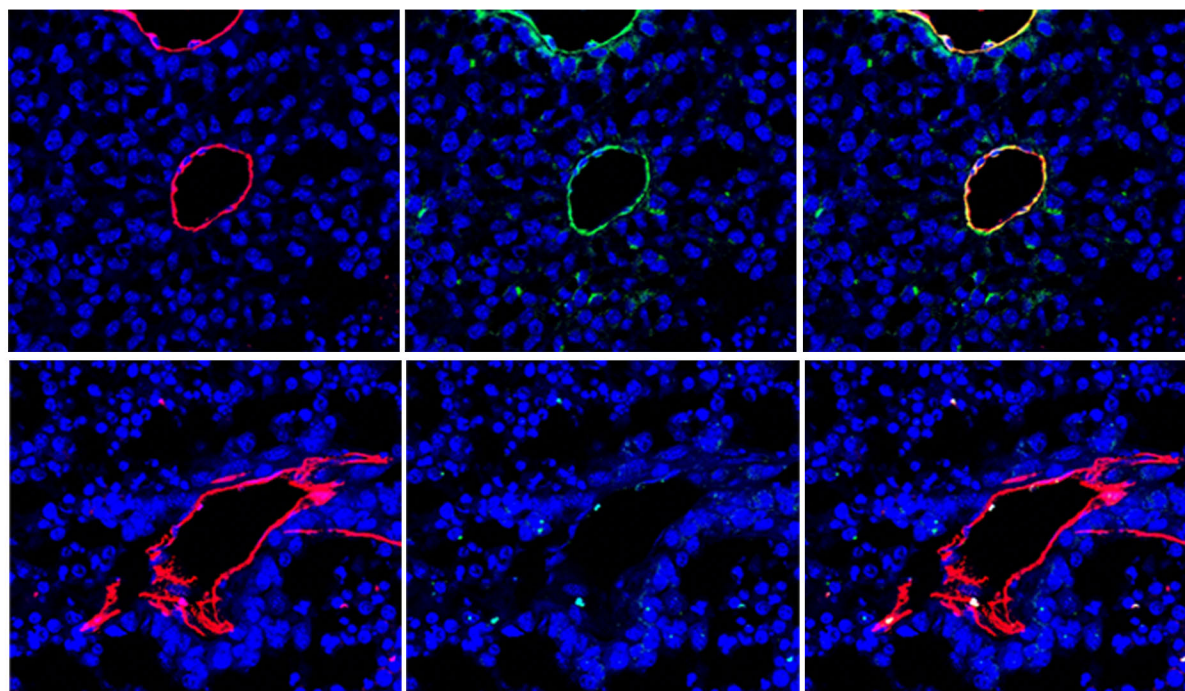

Supplement: Supplementary file 7 — Source Data for Figure 6 [file EMMM-13-e14089-s002.pdf]

Figure 8H

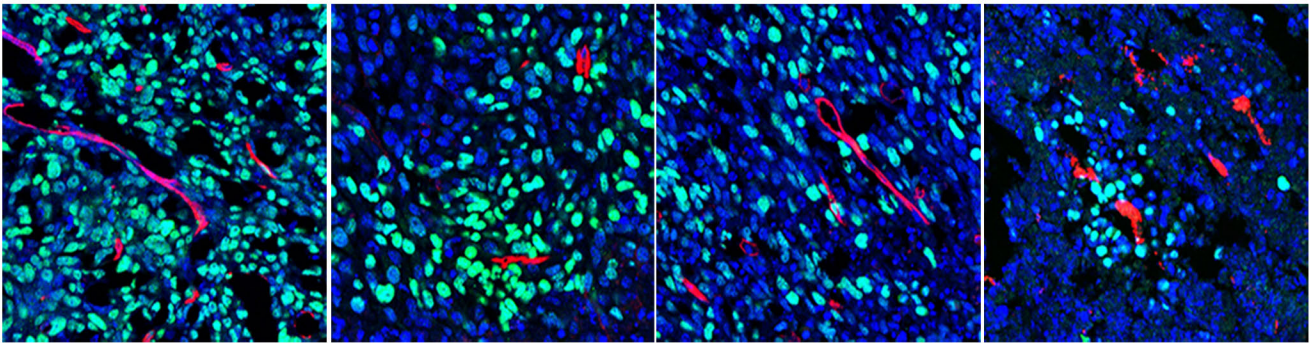

Supplement: Supplementary file 8 — Source Data for Figure 8 [file EMMM-13-e14089-s006.pdf]
